# Ventriculoperitoneal Shunt and Endoscopic Third Ventriculostomy for Hydrocephalus in Adult Patients With Brain Metastases

**DOI:** 10.7759/cureus.77707

**Published:** 2025-01-20

**Authors:** Lisa B Shields, Michael W Daniels, Alexandra Vaynerman, Lennea Coombs, Parag Sevak, Hilary A Highfield, Kaylyn Sinicrope, Aaron Spalding, David Sun

**Affiliations:** 1 Neurological Surgery, Norton Neuroscience Institute, Norton Healthcare, Louisville, USA; 2 Bioinformatics and Biostatistics, University of Louisville, Louisville, USA; 3 Radiation Oncology, Norton Cancer Institute, Norton Healthcare, Louisville, USA; 4 Pathology, Clinical Pathology Accreditation (CPA) Laboratory, Norton Healthcare, Louisville, USA

**Keywords:** brain metastasis, endoscopic third ventriculostomy, hydrocephalus, neuro-oncology, neurosurgery, ventriculoperitoneal shunt

## Abstract

Background: Patients with brain metastases and concurrent hydrocephalus warrant expedited treatment. This study evaluated survival outcomes of patients with brain metastases and hydrocephalus treated with endoscopic third ventriculostomy (ETV) or ventriculoperitoneal shunt (VPS) placement.

Materials and methods: Twenty patients with brain metastases and hydrocephalus were treated with ETV or VPS over 10 years (July 18, 2013-November 20, 2023). Our findings were juxtaposed against data from 77 published controls to assess whether ETV and VPS management correlated with enhanced survival.

Results: The most common primary cancer diagnoses were breast (9 [45%]) and non-small cell lung cancer (5 [25%]). Seven (35%) patients had leptomeningeal carcinomatosis. The initial procedure to treat hydrocephalus was a VPS in 13 (65%) patients; seven (35%) had an ETV first. Patients with a single brain metastatic lesion had a longer median overall survival (OS) than those with more than one metastatic site (154.5 versus 67.0 days). Our cohort had a similar median OS following the ETV/VPS procedure compared to published data (92.5 versus 91 days). In both ETV and VPS subsets, our cohort had a longer median OS than published data: 106 versus 56 days for ETV and 79 versus 56 days for VPS.

Conclusions: Patients with brain metastases and hydrocephalus who underwent an ETV or VPS placement had improved survival compared to historical controls and if they had only one metastatic lesion. Interdisciplinary evaluation of patients with brain metastases by neurosurgeons as well as medical and radiation oncologists is warranted to facilitate systemic therapy after hydrocephalus relief.

## Introduction

Brain metastases are the most common type of brain tumor with an estimated 200,000 cases diagnosed annually in the United States [1]. Approximately 10-40% of adult patients with cancer develop brain metastasis which is most frequent with lung and breast cancers, malignant melanoma, renal cell carcinoma, and colorectal carcinoma [2-6]. The incidence of brain metastasis is escalating due to numerous factors, including increased detection through enhanced MRI techniques, improved control of systemic cancer with immunotherapy, and prolonged survival [1-4]. The treatment of brain metastasis depends on the number and size of the metastatic lesions, control of systemic disease, and a patient’s performance status (Karnofsky Performance Status (KPS) or Eastern Cooperative Oncology Group (ECOG) performance status) [4]. Radiotherapy (whole brain radiation therapy (WBRT) or stereotactic radiosurgery (SRS)), surgical resection, systemic or intrathecal chemotherapy, molecularly targeted therapy, and medical management with corticosteroids or antiepileptic medications are valid options after the diagnosis of brain metastasis [2,4,6,7]. Other patients may select supportive care if they have progressive systemic disease or a poor performance status. 

One-third of patients with breast cancer develop brain metastases which frequently occur in the midline structures of the cerebellum, medulla, and parietal lobe where aromatase is concentrated [8,9]. Estrogen activity in the brain and the local mass effect of the brain metastases are associated with the risk of hydrocephalus [8,9]. Tumor-related hydrocephalus may be the primary cause of the presenting symptoms and often necessitates treatment prior to oncological management. Between 1 and 5% of patients with brain metastasis develop hydrocephalus [10]. Fifty percent of patients will die within three months of treatment for hydrocephalus, and 10% have a one-year survival with systemic disease progression as the leading cause of death [10,11]. Tumor-associated hydrocephalus may result from the overproduction of cerebrospinal fluid (CSF), obstruction of the CSF pathways, or reduced CSF absorption which causes ventricular dilatation and increased intracranial pressure (ICP) [3,12,13]. Hydrocephalus may be either communicating (CSF still flows between the ventricles) or non-communicating/obstructive (partial or complete CSF flow obstruction) [12]. Metastasis to the periventricular brain tissue may obstruct flow of the CSF to the subarachnoid space where it is absorbed by the arachnoid granulations which causes non-communicating hydrocephalus [14]. Symptoms of hydrocephalus include severe headaches, nausea, vomiting, weakness, cognitive dysfunction, cranial nerve palsies, urinary incontinence, visual decline, and gait abnormalities [3,6,11,15-17]. 

Several treatments are available for patients with brain metastasis who experience hydrocephalus such as a ventriculoperitoneal shunt (VPS), endoscopic third ventriculostomy (ETV), repeated lumbar punctures, intraventricular reservoir placement, and pain management without intervention [7,11]. These procedures may be imperative to treat the disabling symptoms since corticosteroids and analgesic medications are often ineffective. Placement of a VPS is the most common procedure and involves a catheter inserted in the ventricle that drains the CSF into the peritoneal cavity where it is absorbed [11,12]. This technique provides rapid normalization of elevated ICP, symptom improvement in 75-90% of patients, and improvements in performance status and quality of life (QOL) [6,11]. These factors encourage VPS use to treat tumor-related hydrocephalus in patients who are poor surgical candidates. However, the complication rate is 10-30%, and several risks have been reported including infection, hemorrhage, shunt malfunction or failure, shunt blockage, subdural collections, and peritoneal tumor dissemination [2,6,11,12,15,17,18]. An ETV is a minimally invasive procedure that involves endoscopic placement of a burr hole at the floor of the third ventricle that permits diversion of the CSF into the interpeduncular cistern [2,12]. This procedure is desirable since it has fewer complications (ventriculitis, meningitis, peritonitis) than VPS [12]. 

Leptomeningeal carcinomatosis (LC), also known as leptomeningeal metastasis, meningeal carcinomatosis, neoplastic meningitis, and carcinomatosis meningitis, is a rare, debilitating complication of advanced cancer where diffuse or multifocal metastasis from a solid tumor infiltrates the leptomeninges with or without brain parenchymal lesions [11,16,17]. Affecting approximately 1-10% of patients with a solid tumor, patients with LC have an average survival of 3.5-6 months [3,6,11,15-19]. Without treatment, the life expectancy after LC diagnosis is 4-6 weeks [15,18]. LC most commonly develops in breast, lung, and ovarian cancers and malignant melanoma [18,19]. Its incidence has increased due to improved diagnostic tools for earlier detection and therapeutic advances to prolong survival. Leptomeningeal spread may obstruct CSF pathways or cause CSF malabsorption leading to increased ICP and hydrocephalus [13,18]. Treatment of LC-associated hydrocephalus depends on a patient’s performance status, neurological deficits, systemic disease control, patient age, and primary tumor histology [11,17,19]. 

We tested the hypothesis that management of hydrocephalus in brain metastasis patients in the era of improved systemic therapy results in increased survival compared to previously reported studies. Secondarily, we tested whether ETV or VPS would be associated with successful management of hydrocephalus in patients with brain metastases. We report 20 patients with brain metastasis and concurrent hydrocephalus who were treated with an ETV or VPS placement. The clinical characteristics and survival analysis of these patients are presented. The benefit of using either an ETV or VPS in patients with brain metastases and hydrocephalus is also discussed. 

## Materials and methods

Under an Institutional Review Board (IRB)-approved protocol and according to the Declaration of Helsinki, we performed a 10-year (July 18, 2013-November 20, 2023) retrospective review of consecutively treated patients with brain metastases and hydrocephalus who underwent either an ETV and/or VPS insertion. Our outcomes were compared to 77 historical controls from the literature [2,10,14,16] to test the hypothesis that management of hydrocephalus in patients who respond to systemic therapy is associated with improvement outcomes. We extracted single patient data points from the cited papers where each patient and their survival were reported individually. We identified the 77 historical controls as they were published as individual data points, specifically each patient's time to survival and time of hydrocephalus free survival. This allowed us to capture each individual data point and do a proper histogram calculation in order to compare to our underlying sample size. Inclusion criteria included adult patients diagnosed with brain metastases by either brain MRI or pathologic confirmation. Patients with LC were not excluded from the study. Patients who declined VPS and ETV were excluded. The decision to perform either an ETV or VPS placement was based on the site of the obstruction. If the obstruction was intraventricular and caudal to the anterior third ventricle, then an ETV was performed. An ETV was preferred over a VPS unless communicating hydrocephalus was present when a VPS was performed. 

Several metrics were collected including age and ECOG status when the brain metastases were diagnosed, gender, site of primary cancer, number and location of brain metastases, communicating versus non-communicating hydrocephalus, procedure to treat hydrocephalus (ETV, VPS, or both), surgical complications, treatment for primary and metastatic tumors, and overall survival (OS). 

Statistical analysis

Counts and percentages for nominal variables and means (with standard deviations) for continuous variables such as age were employed. The median and interquartile range (IQR) described the distribution of brain metastases and survival times. Survival differences were evaluated using the Kaplan-Meier method with log-rank tests. Cox proportional-hazards models were utilized to estimate hazard ratios (HRs) with 95% confidence intervals (CIs). All analyses were performed using R software (v4.3.1 Beagle Scouts; R Foundation for Statistical Computing, Vienna, Austria).

## Results

Clinical characteristics

A total of 20 patients were identified with brain metastasis and concurrent hydrocephalus. The median age was 59.5 [56.0, 65.0] years, and the majority (13 [65.0%)]) of patients were female (Tables 1, 2). Most (13 [65.0%]) patients had an ECOG score of 2 at the time of the initial ETV/VPS procedure. The most common primary cancer diagnosis was breast (9 [45%]) followed by non-small cell lung cancer (5 [25%]). 

**Table 1:** Composite Clinical Characteristics of Patients with Brain Metastasis and Hydrocephalus IQR: interquartile range; VPS: ventriculoperitoneal shunt; ETV: endoscopic third ventriculostomy; LC: leptomeningeal carcinomatosis; OS: overall survival

Features	Category	Number of Patients (n=20)
Age (median [IQR]) at ETV/VPS procedure		59.5 [56.0, 65.0] years
Gender	Male	7 (35%)
	Female	13 (65%)
ECOG score at initial ETV/VPS procedure	0	2 (10.0%)
	1	5 (25.0%)
	2	13 (65.0%)
Primary cancer diagnosis	Breast	9 (45%)
	Non-small cell lung	5 (25%)
	Squamous cell lung	2 (10%)
	Adenocarcinoma of lung	1 (5%)
	Small cell lung	1 (5%)
	Colon	1 (5%)
	Endometrial	1 (5%)
Location of brain metastases	Infratentorial	3 (15%)
	Supratentorial	4 (20%)
	Infratentorial and supratentorial	6 (30%)
	LC	7 (35%)
Number of brain metastases		3.0 [1.0, 10.0]
Type of hydrocephalus	Non-communicating	13 (65%)
	Communicating	7 (35%)
First procedure type after hydrocephalus diagnosis	VPS	13 (65%)
	ETV	7 (35%)
Duration between brain metastases and ETV/VPS procedure (median [IQR])		0.36 [0.18, 2.15] months
OS following primary cancer diagnosis (median [IQR])		20.13 [5.72, 37.98] months
OS following brain metastasis diagnosis (median [IQR])		5.45 [2.39, 8.33] months
OS following initial ETV/VPS procedure (median [IQR])		3.08 [1.42, 5.30] months
OS in ETV subset following ETV (median [IQR])		3.53 [1.23, 5.68] months
OS in VPS subset following VPS (median [IQR])		2.63 [1.53, 5.00] months

**Table 2:** Individual Clinical Characteristics of Patients with Brain Metastasis and Hydrocephalus Supra: supratentorial; Infra: infratentorial; SCLC: small cell lung cancer; SCC: squamous cell lung cancer; NSCLC: non-small lung cancer; LUAD: lung adenocarcinoma; NC: non-communicating; C: communicating; OS: overall survival; ETV: endoscopic third ventriculostomy; VPS: ventriculoperitoneal shunt; LC: leptomeningeal carcinomatosis; *Both: supratentorial and infratentorial

Patient #	Age (Years)/ Gender (M/F)	ECOG Score at Brain Metastasis Diagnosis	# Brain Mets	Location of Brain Mets	Primary Cancer Diagnosis	Hydrocephalus Type (C/NC)	1^st^ Procedure Type (ETV/VPS)	OS following initial ETV/VPS (months)
1	65/M	2	6	Both *	CSLC	NC	ETV	3.53
2	64/F	1	1	Supratentorial	SCC	NC	ETV	16.9
3	47/F	1	13	Infratentorial	Breast	NC	ETV	6.07
4	60/M	1	1	Supratentorial	Colon	NC	ETV	5.3
5	72/M	2	73	Both	NSCLC	NC	ETV	1.83
6	69/M	2	10	Both	NSCLC	NC	VPS	1.53
7	65/M	2	1	Infratentorial	NSCLC	NC	VPS	5.0
8	61/M	1	3	Both	NSCLC	NC	VPS	0.87
9	56/F	2	LC	LC	Breast	NC	VPS	3.77
10	56/F	0	1	Supratentorial	Breast	NC	VPS	32.83
11	57/F	2	LC	LC	Breast	NC	VPS	1.57
12	56/F	1	1	Infratentorial	Breast	C	VPS	2.47
13	69/F	2	10	Both	Endometrial	C	ETV	0.63
14	39/M	2	13	Both	LUAD	NC	ETV	0.50
15	65/F	2	LC	LC	Breast	C	VPS	2.63
16	51/F	2	LC	LC	Breast	C	VPS	4.67
17	44/F	2	LC	LC	Breast	C	VPS	5.53
18	58/F	0	LC	LC	Breast	C	VPS	0.73
19	61/F	2	1	Infratentorial	SCC	NC	VPS	1.07
20	59/F	2	LC	LC	NSCLC	C	VPS	5.30

Of the total 20 patients, seven (35%) had LC. Six of these seven patients with LC had a primary breast cancer pathology. The other patient had primary non-small cell lung cancer. Of the 13 patients without LC, the mean number of metastatic lesions was 9.5 (range: 1-72 lesions). Six (30%) patients had both supratentorial and infratentorial brain metastasis, while four (20%) had infratentorial and three (15%) had supratentorial metastasis. 

The brain metastasis was diagnosed before the primary cancer in 5 (25%) patients and concurrently in one (5%) patient. Of the remaining 14 patients whose primary cancer was diagnosed before the brain metastases, the mean duration of time between the diagnoses was 24.88 months (1.2-74.4 months). 

Procedure to treat hydrocephalus

 A total of 13 (65%) patients with brain metastasis developed non-communicating hydrocephalus, and seven (35%) had communicating hydrocephalus. The median duration between the diagnosis of brain metastasis and ETV/VPS procedure was 0.36 [0.18- 2.15] months. The initial procedure to treat the hydrocephalus was a VPS in 13 (65%) patients; seven (35%) had an ETV first. Three (15%) patients underwent both a VPS and an ETV procedure. One patient experienced an incisional dehiscence of the VPS which necessitated an ETV placement 40 days later. Two other patients initially underwent ETV placement, both of whom had a VPS inserted five days later. Two patients had a VPS revision, and another two had VPS reprogramming. 

Treatments for primary cancer and brain metastasis

Table 3 highlights the treatment for the primary cancer and brain metastasis prior to and following the ETV/VPS procedure. Thirteen (65%) patients underwent treatment after the ETV/VPS. Of these 13 patients, six (46.2%) were treated with WBRT, 5 (38.5%) with palliative systemic chemotherapy, four (30.8%) with intrathecal methotrexate, and two (15.4%) with SRS delivered to the brain. 

**Table 3:** Treatment for Primary Cancer and Brain Metastasis Before and After the ETV/VPS Procedure SCLC: small cell lung cancer; SCC: squamous cell lung cancer; NSCLC: non-small lung cancer; LUAD: lung adenocarcinoma; WBRT: whole brain radiation therapy; SRS: stereotactic radiosurgery

Patient #	Primary Cancer Diagnosis	Treatment before ETV/VPS	Treatment after ETV/VPS
1	CSLC	Ventriculostomy drain	Palliative systemic chemotherapy (carboplatin/VP-16), WBRT
2	SCC	Systemic chemotherapy (opdivo/gemcitabine), SRS (C5 vertebral body)	Palliative systemic chemotherapy (gemcitabine)
3	Breast	None	WBRT (10 fractions, 3000 cGy total dose)
4	Colon	Systemic chemotherapy	None
5	NSCLC	None	WBRT (5 fractions, 2000 cGy total dose)
6	NSCLC	None	WBRT (10 fractions, 3000 cGy total dose)
7	NSCLC	Systemic chemotherapy, radiation to the primary site, craniotomy for metastatic lesion resection, external ventricular drain	SRS brain (1 fraction, 680.3 cGy total dose)
8	NSCLC	Systemic chemotherapy, radiation to the primary site, craniotomy for metastatic lesion resection, SRS brain (three occasions)	None
9	Breast	Systemic chemotherapy	Intrathecal methotrexate, palliative chemotherapy (gemcitabine)
10	Breast	Systemic chemotherapy, craniotomy for metastatic lesion resection, SRS brain (three occasions)	SRS brain (1 fraction, 1619.4 cGy total dose), palliative chemotherapy
11	Breast	Systemic chemotherapy, radiation to the primary site	WBRT (10 fractions, 3000 cGy total dose)
12	Breast	Systemic chemotherapy	WBRT (10 fractions, 1890.4 cGy total dose)
13	Endometrial	Systemic chemotherapy, radiation to the primary site, SRS brain	None
14	LUAD	None	None
15	Breast	Systemic chemotherapy, radiation to the primary site, SRS brain	Intrathecal methotrexate
16	Breast	Systemic chemotherapy, radiation to the primary site, SRS brain (two occasions)	Intrathecal methotrexate
17	Breast	None	Intrathecal methotrexate, palliative chemotherapy
18	Breast	Systemic chemotherapy	None
19	SCC	None	None
20	NSCLC	Systemic chemotherapy	None

Overall survival

All 20 patients with brain metastasis and hydrocephalus died. The median OS following the primary cancer diagnosis was 20.13 [5.72, 37.98] months, and the median OS following the brain metastasis diagnosis was 5.45 [2.39, 8.33] months. The median OS following the initial VPS/ETV procedure was 3.08 [1.42, 5.30] months. The median OS of the patients who only underwent a VPS (12 patients) or ETV (five patients) was 3.53 [1.23, 5.68] months and 2.63 [1.53, 5.00] months, respectively. The median OS following the primary cancer diagnosis (ECOG score 0: 58.2 [33.0, 83.4], ECOG score 1: 28.4 [11.9, 35.7], ECOG score 2: 11.0 [3.7, 33.9], p = 0.511), brain metastasis diagnosis (ECOG score 0: 51.2 [26.0, 76.5], ECOG score 1: 6.2 [5.6, 16.9], ECOG score 2: 4.0 [2.3, 6.0], p = 0.371), and ETV/VPS procedure (ECOG score 0: 16.8 [8.8, 24.8], ECOG score 1: 5.3 [2.5, 6.1], ECOG score 2: 2.6 [1.5, 4.7], p = 0.468) was not statistically significant between ECOG scores. However, patients with an ECOG score of 0 had a longer OS than those with ECOG scores of 1 or 2. 

**Figure 1:**
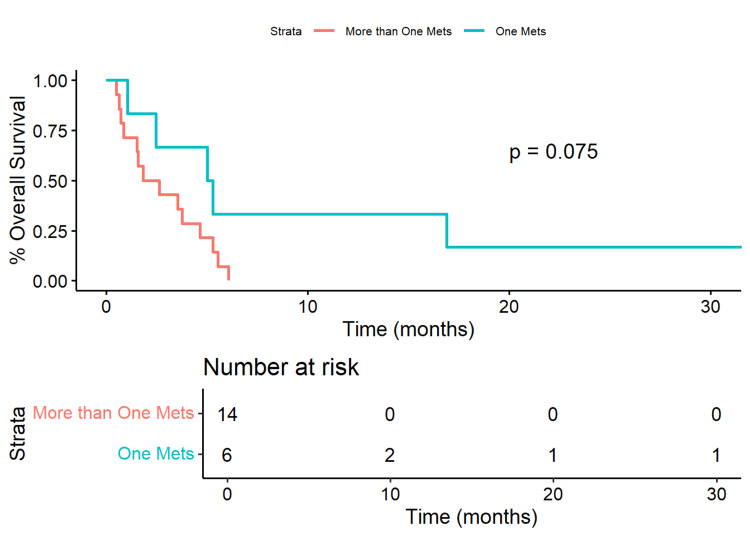
Kaplan-Meier Plot of Overall Survival of Patients with One Metastasis Versus More Than One Patients with only one brain metastatic lesion had a median overall survival of 154.5 (IQR=[93.0,420.0]) days versus those with more than one lesion (median overall survival of 67.0 [IQR=31.0,133.3] days). Patients with only one brain metastasis had a 50% survival time of 154 days versus those with more than one lesion with a 50% survival time of 55 days. Patients with one brain metastatic lesion had a marginally significant 62.4% reduction in risk (HR=0.376, 95% CI=[0.180, 0.787], p=0.089) compared to those with more than one lesion.

In our cohort, patients with a single brain metastatic lesion had a median OS of 154.5 days (IQR=[93.0, 420.0]) compared to those with more than one brain metastasis who had a median OS of 67.0 days (IQR=[31.0, 133.3]). The 50% survival time for patients with one brain metastatic lesion was 154 days compared to 55 days for those with more than one. There was a marginal difference in the survival curves (p=0.075). Patients with one brain metastasis had a marginally significant 62.4% reduction in risk (HR=0.376, 95% CI=[0.180, 0.787], p=0.089) compared to those with more than one brain metastasis (Figure 1). 

**Figure 2:**
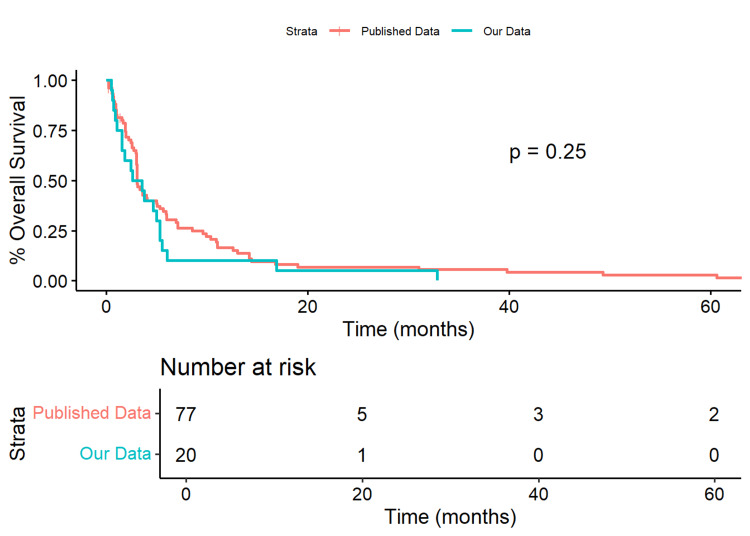
Kaplan-Meier Plot of Our Cohort Versus Published Data Our data had a median overall survival of 92.5 (IQR=[42.5,159.0]) days compared to 91 (IQR=[50.0,212.0]) days in the published data (Wilcoxon Rank Sum p=0.527).  Our data had a 50% survival time of 79 days, while the published data was 91 days.  Our cohort had an insignificant 33% increase in risk (HR=1.328, 95%CI=[0.805, 2.191], p=0.267) compared to the published data.

Our cohort had a median OS of 92.5 days (IQR=[42.5, 159.0]) compared to 91 days (IQR=[50.0, 212.0]) in the published data (Wilcoxon Rank Sum p=0.527). The 50% survival time for our data was 79 days, while it was 91 days in the published data. Our cohort showed a non-significant 33% increase in risk (HR=1.328, 95% CI=[0.805, 2.191], p=0.267) compared to the published data (Figure 2).

**Figure 3:**
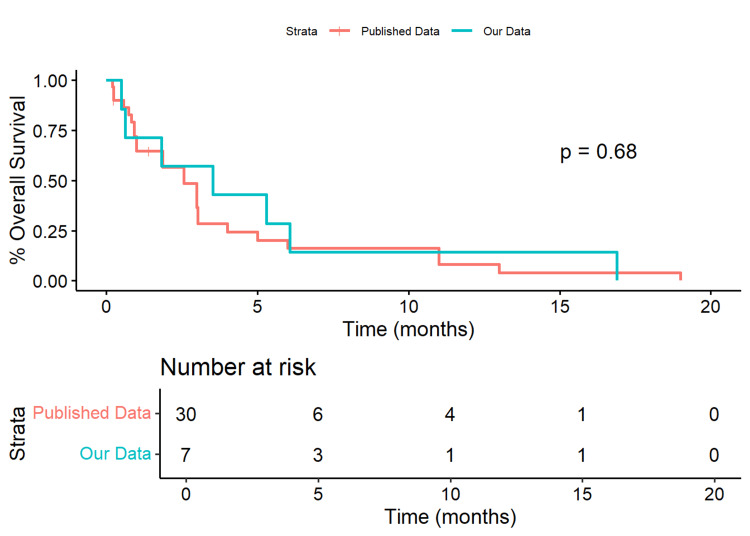
Kaplan-Meier Plot of Our Cohort Versus Published Data for ETV Only When evaluating a subset of ETV procedures only, our data had a median overall survival of 106 (IQR=[37.0,170.5]) days compared to 56 (IQR=[25.8,91.0]) days in the published data (Wilcoxon Rank Sum p=0.382).  Our data had a 50% survival time of 106 days, while the published data was 77 days.  Our cohort had an insignificant 19% decrease in risk (HR=0.814, 95%CI=[0.348, 1.903], p=0.635) compared to the published data. ETV: Endoscopic third ventriculostomy

In the subset of ETV procedures, our data had a median OS of 106.0 days (IQR=[37.0, 170.5]) compared to 56 days (IQR=[25.8, 91.0]) in the published data (Wilcoxon Rank Sum p=0.382). The 50% survival time for our data was 106 days, while it was 77 days in the published data. Our cohort had a non-significant 19% decrease in risk (HR=0.814, 95% CI=[0.348, 1.903], p=0.635) compared to the published data (Figure 3).

**Figure 4:**
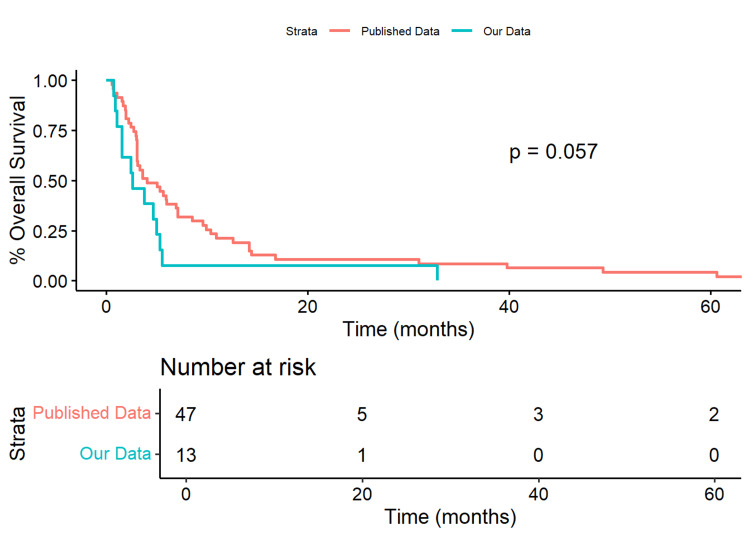
Kaplan-Meier Plot of Our Cohort Versus Published Data for VPS Only When evaluating a subset of VPS procedures only, our data had a median overall survival of 79 (IQR=[46.0,150.0]) days, while the Published Data was 56 (IQR=[25.8,91.0]) days (Wilcoxon Rank Sum p=0.074). Our data had a 50% survival time of 79 days, while the published data was 121 days.  Our cohort had a marginally significant 81.5% increase in risk (HR=1.815, 95%CI=[0.966, 3.412], p=0.064) compared to the published data. VPS: Ventriculoperitoneal shunt

For the VPS procedures, our data showed a median OS of 79.0 days (IQR=[46.0, 150.0]) compared to 56 days (IQR=[25.8, 91.0]) in the published data (Wilcoxon Rank Sum p=0.074). The 50% survival time for our data was 79 days, while it was 121 days in the published data. There was a marginal difference in the survival curves (p=0.057). Our cohort showed a marginally significant 81.5% increase in risk (HR=1.815, 95% CI=[0.966, 3.412], p=0.064) compared to the published data (Figure 4).

## Discussion

Few studies have reported the use of ETV and VPS in patients with brain metastasis who develop hydrocephalus [2,3,7,10,16,17,19]. In Jung and colleagues’ study of 1343 patients with brain metastasis from systemic solid tumors, 71 (5.3%) had LC [19]. Eighteen (25.4%) of these 71 patients had evidence of hydrocephalus, seven of whom underwent a VPS insertion. Patients with surgically untreated and treated hydrocephalus demonstrated a median OS of 1.7 and 5.7 months, respectively. These authors proposed that functional status and adjuvant local and systemic treatment should be considered to improve survival in LC patients who undergo surgical treatment of hydrocephalus [19]. In Yoshioka and colleagues’ study of 14 patients with LC-associated hydrocephalus who underwent VPS placement, symptoms and KPS improved in all patients [7]. The median OS was 3.7 months, although two patients who underwent molecular targeted therapy after the VPS shunt survived for more than one year. In Lee and colleagues’ study of 50 patients with brain metastasis (10 with parenchymal metastasis and 40 with LC) who developed hydrocephalus and had a VPS insertion, the median OS from the surgery was three months (2 days-54 months) [3]. Similarly, the studies of Murakami et al. and Omuro et al. on LC patients with hydrocephalus who were treated with a VPS reported improved symptoms and an improved OS after VPS placement of 3.3 and 2 months, respectively [16,17]. Murakami and colleagues concluded that VPS surgery is an effective palliative option for patients with a KPS of more than 30 as it provides an enhanced QOL [16]. 

In the studies by Gonda et al. and Chen et al. on patients with brain metastasis who had either a VPS (36 patients) [10] or ETV (16 patients) [2] and patients who had more severe hydrocephalic symptoms were more likely to benefit from ETV and VPS [2,10]. Patients with no history of infection or ventricular hemorrhage and no evidence of LC were treated with an ETV; the remainder had a VPS. Of the 36 patients who underwent VPS, nine had LC. The median OS was three months for ETV and 5.5 months for VPS. Nine (25%) patients who had a VPS survived more than one year, while two survived more than five years. The complication rate was comparable between ETV (12.6%) and VPS (19.4%). Seven shunt failure or shunt-related complications that required further surgery were noted in the VPS group. One patient who had an ETV experienced a postoperative wound infection requiring debridement, and another underwent external ventricular drainage for assessing ICP. 

In Pasqualotto and colleagues’ systemic review and meta-analysis of patients with obstructive hydrocephalus who underwent either an ETV or VPS, 2353 studies were identified, including five randomized clinical trials with 310 patients [12]. A total of 163 patients underwent an ETV. ETV had significantly fewer postoperative infections than VPS, although there was no significant difference for postoperative or intraoperative hemorrhage, postoperative CSF leak, operative success, and mortality. There was a significantly higher incidence of complications in patients who underwent a VPS. 

Our study concurs with the extant literature with respect to the OS following the ETV/VPS procedure. The median OS after an ETV/VPS of our cohort was 92.5 days compared to 91 days in the literature (p=0.527) [2,10,14,16]. Both the ETV and VPS subsets in our study had a longer median OS compared to the literature, with 106 versus 56 days in the ETV group and 79 versus 56 days in the VPS group. Additionally, our study corresponds to the literature about the rare and minimal complications associated with the ETV/VPS procedure in patients with brain metastasis. Only one (5%) patient experienced a complication in our work, specifically, incisional dehiscence after a VPS. This complication percentage is lower than that reported by Gonda et al. and Chen et al.: 12.6% for ETV and 19.4% for VPS [2,10]. 

Strengths and limitations of the current study

The strength of the present study is the large number of patients with brain metastases and hydrocephalus who underwent a VPS/ETV placement. This study adds to the burgeoning literature about the safety and efficacy of VPS/ETV in treating patients with brain metastases and hydrocephalus. Neurosurgeons should consider the value of these procedures in prolonging survival when evaluating patients in the Emergency Department with hydrocephalus. The limitation of the current study is its retrospective nature as well as the small cohort size. We were not blinded to the results of the historical control group, although our treatment team was not all aware of these previous patient data at the time of treatment decisions. We agree that a true randomized study would be ideal with stratifications between the treatment groups prospectively determined. Our current study attempted to use these historical controls as a comparison rather than publish a descriptive outcome measurement of our current patient outcomes. Additionally, with a sample size of 20 patients, a power calculation with an alpha error of less than 5%. There is a 50% probability to detect a 20% difference in the rate of successful hydrocephalus treatment. It would take a sample size of 35 patients to have an 83% probability. Thus, we continue to collect data on similar patients and will test this hypothesis again when additional patients are available.

## Conclusions

Patients with brain metastases and associated hydrocephalus require expedited treatment. Survival outcomes of patients with brain metastases and hydrocephalus treated with ETV or VPS placement were assessed. Patients with brain metastases and hydrocephalus who underwent an ETV or VPS placement had improved survival compared to historical controls and if they had one metastatic lesion. A high index of suspicion is warranted for metastatic breast and lung cancer and malignant melanoma when patients present with symptoms of hydrocephalus. The use of shunting can be an important tool to allow patients to pursue additional treatments such as systemic or radiation therapy.
